# Digital and plasmonic artificial neural networks—Improved nonlinear signal processing at high speed and low complexity

**DOI:** 10.1126/sciadv.adx1657

**Published:** 2025-11-14

**Authors:** Tobias Blatter, Amane Zürrer, Yannik Horst, Christos Pappas, George Giamougiannis, Apostolos Tsakyridis, Wolfgang Heni, Manuel Kohli, Ueli Koch, Miltiadis Moralis-Pegios, Nikos Pleros, Juerg Leuthold

**Affiliations:** ^1^ ETH Zurich, Institute of Electromagnetic Fields (IEF), Gloriastrasse 35, 8092 Zürich, Switzerland.; ^2^ Department of Informatics, Aristotle University of Thessaloniki, 54124, Thessaloniki, Greece.; ^3^ Polariton Technologies AG, 8134 Adliswil, Switzerland.

## Abstract

Transmission at ever higher data rates increasingly demands more advanced digital signal processing techniques, raising both power consumption and operational costs. Here, we introduce a photonic/plasmonic artificial neural network (ANN) using plasmonic modulators to directly mitigate nonlinear signal distortions carried by an optical carrier. This first-of-its-kind plasmonic ANN achieves an ultracompact footprint and high-speed operation and markedly reduces the need for electronic processing. We compare our plasmonic ANN against a traditional digital feed-forward equalizer and a Volterra series, as well as the corresponding digital ANN. The results demonstrate that an astonishingly small ANN outperforms classical equalizers by attaining higher SNR at smaller computational effort. While the digital ANN offers an ideal implementation, executing the ANN on our first plasmonic chip already shows remarkable equalization performance with minimal components. The findings reveal a path toward ultracompact, high-speed, power-efficient, low-latency alternatives to conventional signal processing.

## Teaser

Plasmonic technology is opening the door to ultrafast and compact analog signal processing for future generations.

## INTRODUCTION

The prevailing strategies for mitigating linear and nonlinear signal distortions in optical communication systems primarily use digital signal processing (DSP) techniques. For their implementations, dedicated advanced electronic components need to be used to accommodate the ever-increasing data transmission rates. Now, up to 50% and more of the total energy consumption in an optical link can be attributed to the DSP electronics, consequently increasing operational costs (*1*, *2*). Toward this end, a multitude of DSP algorithms pursuing different techniques has been developed. The question is which technique will provide the highest improvement of a degraded signal at the lowest complexity. The important figures of merit are the signal-to-noise ratio (SNR) and the required number of multiplications.

Established DSP algorithms include, for example, feed-forward equalizer (FFE), Volterra (VLT) series, and digital backpropagation. Artificial neural networks (ANNs) offer an alternative approach to the established DSP algorithms (*3*). Yet, ANNs have, in general, a reputation for being energy hungry (*4*). Recent investigations have indicated the potential of digital ANNs (DNNs) for DSP to reduce computational power demands (*5*–*7*). An ANN relies heavily on multiplication and accumulation (MAC) operations. Since such MAC operation can be performed easily in the optical domain, photonic neural networks are emerging as an alternative to DNN (*8*, *9*). Unlike electrical signals, optical signals can carry massive bandwidths with minimal latency. In a photonic system, multiple optical signals can be combined, split, and weighted simultaneously, enhancing processing efficiency (*10*–*12*). In many schemes, the weights are adjusted infrequently, allowing them to be treated as static. As a result, energy consumption remains low, even as symbol rates increase. Moreover, the weighting process itself can be highly energy efficient, e.g., see (*13*, *14*). Consequently, photonic platforms as an alternative to the digital approach have recently drawn attention. Those platforms promise not only an increase in computation speed but also a substantial reduction in power requirements (*10*, *15*–*23*).

**Fig. 1.**
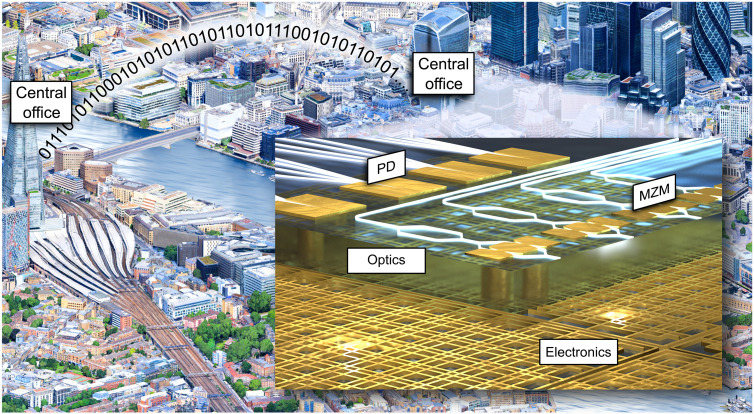
Device and application. The image illustrates two connected transceivers in central offices. In the central offices, receivers relying on plasmonic ANNs may directly process optical signals independent of the bandwidth. The concept is shown in the inset: The chip consists of a photonic and an electronic layer stack. The photonic stack hosts the optical MAC schemes and highspeed, ultracompact plasmonic modulators. The electronic stack hosts some of the nonlinear activation functions and is needed to control the linear operations in the optical stack. Also, it serves as an electronic data interface to the chip.

While the linearity of light brings the opportunity to increase speed and energy efficiency in MAC operations, the linearity also makes it challenging to implement an all-optical nonlinear activation function (*24*). One promising approach relies on the combination of analog electronics and photonics; see the wider vision illustrated in Fig. 1. In this approach, a PD transforms the photonics signal to the electrical domain, where an activation function is applied. Afterward, a modulator maps the activation function’s output back on a photonic signal. A disadvantage of such electro-optical components is the usual bandwidth and footprint limitation that goes along with such components. However, when using plasmonic instead of photonic components, one could take advantage of both the PDs and modulators’ unprecedented speed and compactness, decreasing latency and footprint requirements. It has been shown that plasmonic-graphene PDs (*25*) and plasmonic-organic (PO) modulators (*26*) have bandwidths exceeding 500 GHz. In addition, they offer a very compact design in the order of 10 μm (*27*, *28*) and low energy consumption in the order of atto-joules per bit (*29*). They create a future-proof highspeed bridge between the electrical and photonic domains, allowing to combine the benefits of the photonic system and the electrical activation (*30*).

Combining the benefits of DNNs with the benefits of photonic computing promises an even further reduction in power consumption alongside enhancements in processing speed. As an example, it has been shown in simulation that photonic neural network platforms have the potential to efficiently undo fiber nonlinearities (*31*). However, in this example, the achieved speeds cannot match those of today’s symbol rates, as it requires digital feature extraction, ultimately leading to an increase in latency. In particular, in data centers, low latency is of the essence. Therefore, a natural way to achieve a benefit is by ANN-inspired signal processing directly on the analog optical signal. This technique has been recently demonstrated by processing optical signals at data rates of 16 Gbit/s (*32*) and 40 Gbit/s (*33*) using photonic perceptrons. Yet, these photonic perceptrons are linear ANNs, which, as such, can only mitigate linear signal distortions.

Here, we compare the signal processing performance of traditional FFE and VLT with the performance of a DNN and a plasmonic neural network (PNN). These equalizers were tested by measuring the SNR after processing a nonlinearly distorted signal. Alongside this, the complexity in terms of the number of multiplications required by those methods was determined. Our findings indicate that an already astonishingly small DNN (one hidden layer with four neurons) outperforms the traditional FFE and VLT with much lower computational complexity. This complexity can be even further reduced by a PNN. Toward this end, to our knowledge, we have implemented the first PNN partially executing an ANN that is trained for channel equalization by operating directly on the received signal. The PNN is based on plasmonic Mach-Zehnder modulators (MZMs). Plasmonics offers a large bandwidth, a low energy consumption, and a compact design. These properties give PNNs the potential to boost the computational speed while simultaneously decreasing the footprint by an order of magnitude compared to traditional photonic modulation methods. In this first experimental implementation, the PNN processed signals at a line rate of 48 Gbit/s and achieved higher SNR values than the digital linear filter. This work extends the contribution presented at the Optical Fiber Communication Conference 2024 (*34*).

## RESULTS

### Comparison of traditional versus neural network signal processing in communications

We have compared five digital and a photonic signal processing methods at the receiver side of an intensity modulation (IM) with direct detection (DD) link for their ability to increase the SNR and reduce the computational complexity. In this experiment, the signal processing techniques are needed to overcome the nonlinear distortions originating at the transmitter. The nonlinear distortions stem from an radio frequency (RF) amplifier that drives an electro-optic MZM, which modulates the intensity of an optical field.

**Fig. 2.**
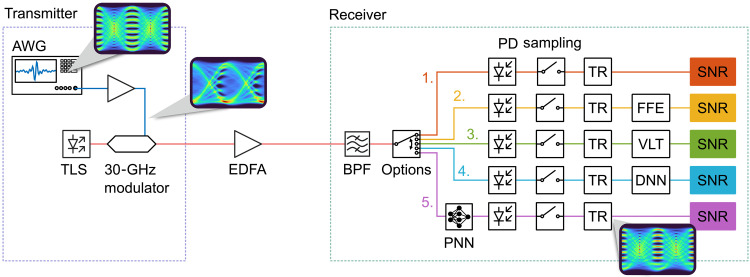
Communication link with processing methods. Experimental setup consisting of a transmitter (left) and a receiver (right). At the transmitter, the RF signal from the AWG is amplified by an RF amplifier, which distorts the signal nonlinearly. The amplified signal is used to drive an MZM with a 3-dB bandwidth of 30 GHz, which modulates the intensity of the optical field that stems from a tunable laser source (TLS). The optical signal is then amplified and filtered. At the receiver side, one of five options can be selected. In the upper four options, the signal gets first detected by a PD, sampled and fed to the TR. In option two to four, the signal is subsequently processed either by a FFE, a VLT equalizer or a DNN. In the last option, the optical signal is processed by a PNN before the direct-detection to reconstruct the original signal. BPF, bandpass filter.

The experimental setup is shown Fig. 2. Uniformly distributed bits were encoded to M-pulse amplitude modulated symbols. An arbitrary waveform generator (AWG) provided the corresponding electrical data signal. This signal was amplified and hereby nonlinearly distorted. The eye diagrams in the electrical domain illustrate this distortion. The amplified signal then drove a commercial MZM with a 3-dB bandwidth of 30 GHz, which modulates the intensity of the optical field, providing an amplitude-shift keying (ASK) signal in the optical domain (8-ASK). After amplification with an erbium-doped fiber amplifier (EDFA) and filtering with a bandpass filter, the signals were fed in either of five receiver schemes:

1) In the first option (red path), the signal is received by a photodiode (PD), sampled, and then fed into the timing recovery (TR).

2) In the second to fourth options, the sampled PD signal is fed after the TR to a FFE,

3) a third-order VLT equalizer or

4) a DNN consisting of one hidden layer featuring four neurons.

5) In the fifth option, the signal is processed in an analog manner on a PNN before being detected by the PD. The PNN implements the same architecture and weight as the digital twin, i.e., the DNN.

More precisely, in the common path of all the receivers, the intensity signal was converted back to the electrical domain using a PD. Subsequently, the electrical signal was sampled using a real-time oscilloscope. The sampled data were then fed to a processing line comprising a TR, which was followed by either of the above-mentioned FFE, a VLT, or a DNN digital processing scheme. The PNN of option five is described in more detail in the “Plasmonic neural network” section below.

**Fig. 3.**
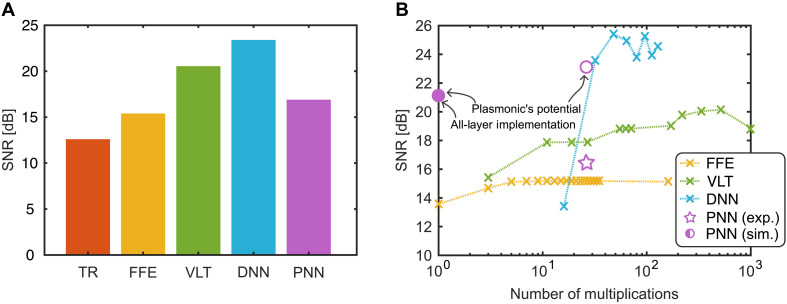
Experimental results. (**A**) Comparison of achievable SNR values after using TR only, TR in combination with FFE, VLT, and a DNN. The application of a PNN provided an SNR that outperformed the traditional FFE DSP. (**B**) Complexity comparison of the digital processing methods. The complexity is quantified by the required number of multiplications (*x* axis), and the performance is measured in terms of SNR (*y* axis). The SNR of the FFE (yellow) saturates with as few as five multiplications. Yet, its SNR remains low. Applying a VLT DSP can further increase the SNR compared to the FFE. The DNN outperforms all other schemes, with delivering a high SNR (>20 dB) with very few multiplications. Using a scheme with plasmonic accelerator allows to decrease the number of multiplications (pink star). Nonidealities in the fabrication led to lower SNR than what would be possible (pink circle). Simulations with experimental data indicate as of what potentially would be possible if all layers were implemented in plasmonic/photonic platform (pink full circle).

The performance of the signal processing methods was measured in terms of the SNR. The TR results also served as a reference baseline. Figure 3 summarizes our findings on the processing performance: Fig. 3A shows the SNR of a signal carrying 48 Gbit/s encoded to an pulse amplitude modulation (PAM) format with 8 levels (8PAM) after undergoing TR signal processing only (red), after TR processing followed by FFE (yellow), after TR and VLT processing (green), and after TR and DNN processing (blue). It can be seen that the DNN outperforms the standard DSP methods. Specifically, after the DNN, the SNR is about 2 dB better compared to the VLT. When executing the DNN on the analog PNN (violet), the performance decreased compared to its digital counterpart. However, the photonic accelerator PNN still outperforms the FFE by 1.5 dB.

The DNN not only outperforms the standard methods in terms of SNR but also does it at a lower complexity as shown in Fig. 3B. The plot shows the achievable SNRs for a given number of digital multiplications for the FFE (yellow), VLT (green), and the DNN (blue) processing schemes. The parameters for each equalizer have been swept and adapted to the optimum value for a given number of multiplications. For instance, one can see to what extent an increase in the number of multiplications improves the SNR. This improvement comes at the price of higher computational effort due to more multiplications. More multiplications give the equalizer or neural network more degrees of freedom, e.g., number of filter taps or number of neurons, which helps to better compensate for distortions. For the VLT, we performed a full sweep of all possible taps in the three orders and then plotted the best SNR results for a given number of multiplications. For the DNN with three layers, we swept the input layer size while keeping the number of neurons in the hidden layer at four (see in-depth discussion below). This results in a small network. It can be seen how the DNN outperforms the FFE and VLT in terms of SNR with an impressively low number of multiplications. To achieve a high SNR (greater than 20 dB), the DNN required substantially fewer multiplications than the VLT equalizer. Specifically, the DNN needs only seven input samples and four hidden neurons to exceed the 20-dB threshold.

### Signal processing methods

In the following, we will delve deeper into the details of the signal processing methods that have been used.

#### Feed-forward equalizer

The FFE is a linear filter used to undo linear distortion in the channel. In essence, the FFE implements the convolution with its weights in Eq. 1.

$$y_{\text{FFE}}\left[n\right]=\sum_{i=0}^{N_{1}-1}w_{i}x\left[n-i\right]$$ (1)

Here, $w_{i}$ represents the value of the *i*th tap (weight) that is multiplied by the sampled signal x[n−i]. The weights were found by minimizing the mean squared errors between the 10% transmitted and received signal. In total, $N_{1}$ multiplications, one per tap, are required to calculate the nth output $y_{\text{FFE}}\left[n\right]$ (*35*).

#### Volterra series

The VLT series can compensate for nonlinear distortions, similar to the Taylor series. In contrast to the Taylor series, the VLT series can also model memory effects. The output of the third-order VLT can be written as

$$y_{\text{VLT}}\left[n\right]=y_{\text{VLT}}^{\left(1\right)}\left[n\right]+y_{\text{VLT}}^{\left(2\right)}\left[n\right]+y_{\text{VLT}}^{\left(3\right)}\left[n\right]$$ (2)

where $y_{\text{VLT}}^{\left(1\right)}\left[n\right]$ is the linear part similar to the FFE, i.e.

$$y_{\text{VLT}}^{\left(1\right)}\left[n\right]=\sum_{i=0}^{N_{1}-1}w_{i}x\left[n-i\right]$$ (3)

and $y_{\text{VLT}}^{\left(2\right)}\left[n\right]\;\text{and}\;y_{\text{VLT}}^{\left(3\right)}\left[n\right]$ are the second- and third-order parts, respectively (*36*). The second-order part is calculated by

$$y_{\text{VLT}}^{\left(2\right)}\left[n\right]=\sum_{j=0}^{N_{2}-1}\sum_{i=0}^{N_{2}-1}w_{\mathrm{ij}}x\left[n-i\right]x\left[n-j\right]$$ (4)

which composes of a total of $2N_{2}^{2}$ multiplications. However, since the system is causal, the tap values are symmetrical, i.e., $w_{\mathrm{ij}}=w_{\mathrm{ji}}$. This allows to reduce the number of multiplications, c.f. (*37*), to

$$2\left(\begin{array}{c} N_{2}+1\\ 2 \end{array}\right)$$ (5)

The third-order part is calculated by

$$y_{\text{VLT}}^{\left(3\right)}\left[n\right]=\sum_{k=0}^{N_{3}-1}\sum_{j=0}^{N_{3}-1}\sum_{i=0}^{N_{3}-1}w_{\mathrm{ijk}}x\left[n-i\right]x\left[n-j\right]x\left[n-k\right]$$ (6)

The third-order part includes a total of $3N_{3}^{3}$ multiplication for each output. However, as the product of $x\left[n-i\right]x\left[n-j\right]$ is already calculated on the second order sum, the number of multiplications reduces to $2N_{3}^{3}$. If one also exploits the symmetries, the number of multiplications can be lowered to

$$2\left(\begin{array}{c} N_{3}+2\\ 3 \end{array}\right)$$ (7)

Similar to the case of the FFE, the weights were found by minimizing the mean squared error of the transmitted and received signal. Thereby, 10% of the recorded signal was used to find the weights.

#### Digital artificial neural network

The chosen DNN architecture is a time-delay neural network executing a regression task. In the regression task, the received symbol blocks is mapped to one transmitted symbol. This network was trained using a subset of the experimentally acquired data within the experiment shown in Fig. 2. In this experiment, the transmitted bits were randomly generated to avoid overfitting that may occur with pseudo-random bit streams (*38*). After recording the symbols and recovering the timing, 10% of the recorded symbols were then used to train the DNN, and the remaining set was used to evaluate the DNN. Further details are reported in Materials and Methods.

**Fig. 4.**
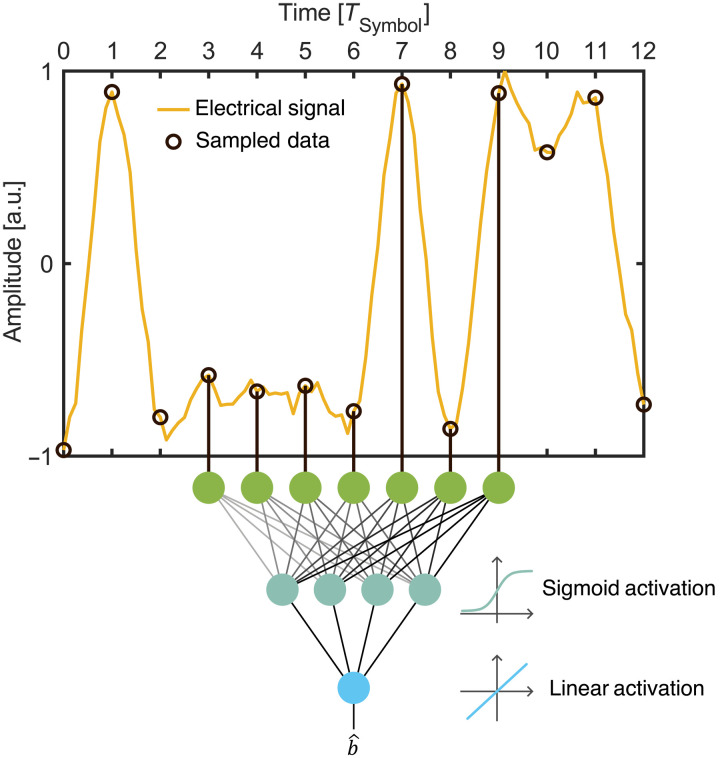
Neural net architecture. Schematic concept of the time-delayed neural network. The electrical signal (yellow) is sampled (black) and fed blockwise into the input layer (green circles) and fed forward to the output. In the hidden layer (green-blue), a sigmoid activation function is used. A linear activation is used at the output layer (blue circle). As a result, the network estimates directly the symbol in the middle of the block. a.u., arbitrary units.

The architecture concept of the network is illustrated in Fig. 4. The sampled symbols (black ring) of the underlying electrical signal (yellow line) are fed into the input layer (green circles). The input layer with P input nodes is fully connected to the hidden layer with four neurons (green-blue circles). At the hidden layer, the weighted ($w_{\mathrm{ij}}$) signals from the input layer are added to a bias term $c_{i}$ and fed into a sigmoid function that served as the activation function σ. Its outputs are then forwarded to the output layer. Here, the weighted ($h_{i}$) signal are again added and mapped to the linear neuron (light-blue circles), giving an estimation $\hat{b}$ of the transmitted symbol b, i.e.

$$\hat{b}\left[n\right]=\sum_{i=1}^{H}h_{i}\sigma \left(\sum_{j=1}^{P}w_{\mathrm{ij}}x\left[n-j\right]+c_{i}\right)$$ (8)

The number of multiplications required to calculate all preactivation values of the fully connected DNN that has P input nodes, H hidden layer nodes, and one output node is

P·H+H (9)

Within this study, we neglected the impact of the activation function on the complexity. Further investigation’s on the fundamental benefit of the DNN over the VLT can be found in the Supplementary Materials. Therein, we followed the work of (*39*).

#### Plasmonic neural network

**Fig. 5.**
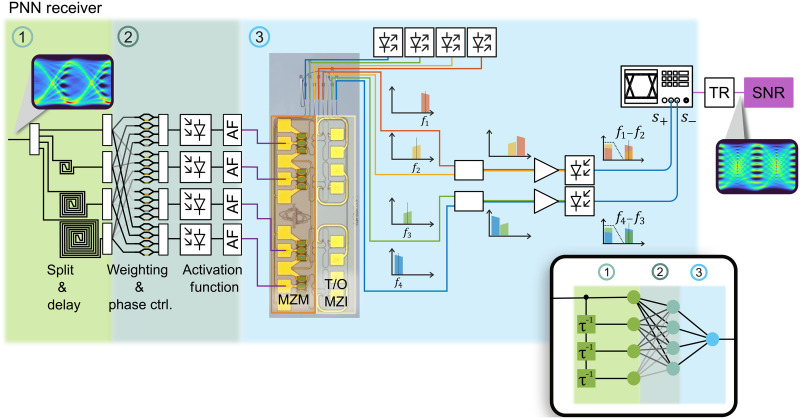
PNN receiver concept. The optical signal after the channel directly enters an on-chip split and delay section. Subsequently, the signal undergoes weighting and phase adjustments to be acquired in a PD. An activation function (AF) is then applied on the electrical signal. Experimentally, the outcomes were computed offline using the experimental data. The resulting signals drive the four plasmonic MZMs (highlighted in orange), modulating the carrier’s intensity. The weight imprinting is handled by the T/O MZM (highlighted in yellow). After merging, the weighted sum is captured. The weight’s sign is preserved by separately recording the sum corresponding to that weight sign, i.e., $s_{+}$ and $s_{-}$. The difference between and $s_{+}$ and $s_{-}$ was calculated offline and correspond to the ANN’s output.

This section conveys the vision of a fully analog photonic neural network and shows a practical implementation mimicking said PNN (accelerator) network with the assistance of electrical activation. The PNN is envisioned to execute the DNN calculations in the optical domain. Thereby, the optical signal that arrives at the receiver is fed to the photonic chip, illustrated in Fig. 5.

The first part of the neural network comprises of the input layer (1) in green and the hidden layer (2) shown in green-blue. The input layer (1) has seven nodes and is implemented on a photonic chip which performs a series of operations (remark: here, we only show four nodes for the sake of simplicity). The incoming data signal is directly split up and delayed with respect to each other. Then, in the hidden layer (2) with four nodes, the weights of the input layer are imprinted on copies of the delayed signals with electrically controlled Mach-Zehnder interferometers (MZIs). The MZIs are equipped with offset phase shifter to also control the phase between the signals, allowing coherent detection. Afterward, the weighted signals are recombined to form the sum required for the hidden layer’s activation function. Then, PDs convert the optical signal to an electrical signal. The signals are subsequently fed into an electrical driver that, in this vision, features an amplification and the activation function (*40*). Alternatively, the activation function can also be provided by any of the schemes listed in reference (*24*), e.g., the subsequent electro-optical modulator itself (*41*) or memristive devices (*42*).

The third layer, i.e., the output layer (3), features one linear neuron. The resulting electrical signals from the hidden layer drive electro-optical MZMs, which take care of the transition back into the optical domain. The weights of the hidden layer are then again imprinted with MZIs. After combining in the optical domain, the optical signals are fed to two PDs to form the sum. Two PDs are used, one for the negative and the other for the positive weights. That way, the sign of the weight is preserved during square-law detection in the PD. In the last step, the two signals are used to directly generate the estimated symbol.

Within this study, the input and hidden layer were implemented offline. Specifically, the four hidden layer outputs were calculated offline and then used to drive the output layer, which was implemented in the experiment. The output layer consisted of PO MZMs (*29*, *43*) and thermo-optical (T/O) MZIs. The output of layer 2 was fed into the MZMs to map the electrical signals back to the optical domain. The fast frequency response helps to encode the electrical signal without any degradation of the laser signal. The signals from the MZM were then fed into T/O-controlled MZI, which provided the weighting (trained as described above). The MZMs and MZIs are highlighted in orange and yellow in the microscope image in Fig. 5, respectively. One wavelength was selected for each MZM to reduce coherent effects in the last PD, i.e., such that the mixing terms at $f_{1}-f_{2}$ and $f_{4}-f_{3}$ are much larger than the PD bandwidth. In addition, the sign of the weights was preserved by combining all optical fields corresponding to either the positive or negative sign and feeding them into the respective input of a balanced PD. Since the signals at different wavelengths are grouped and routed to fixed detector inputs, the signs of the weights can only be changed by rerouting the signals to different ports. In our experiment, there was no need to change signs after offline training. Yet, future implementations can allow more flexibility by alternative sign preservations, e.g., (*44*), or by adapting the training procedure, e.g., (*45*). The subtraction can be done within a balanced PD; however, in this study, we recorded the two signals separately and subtracted the signals digitally. The performance when executing the DNN on the PNN is discussed in the next section.

### Comparison between DNN and PNN processing

In this section, we report on the performance when executing the DNN on the PNN. The experimental setup with the chip, as described in the “Plasmonic neural network” section, was implemented as follows: The input layer and hidden layers were calculated offline, while the output layer was executed in the optical domain. Figure 5 shows a microscope image of the fabricated chip (gray area in the center), which is part of the output layer. It consists of four plasmonic MZMs (highlighted in orange), each of which is connected to a T/O MZI (highlighted in yellow), which in turn imprints the weight. The chip was fabricated on Polariton Technologies’ SiPh-Plasmonic PIC platform. Further details on the fabrication can be found in (*46*–*48*).

**Fig. 6.**
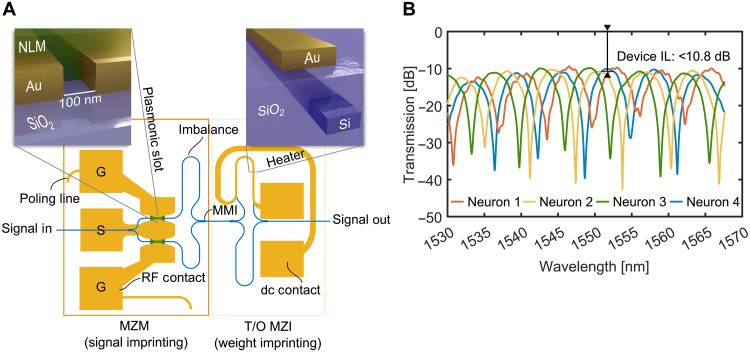
MZMs’ transmission spectrum. (**A**) Schematic of one of the four plasmonic neurons. It consists of weight imprinting plasmonic MZM and a weight imprinting T/O MZM and the Si routings between them. The insets depict a visualization of the plasmonic slot and the heater. It also shows the contacts for the RF and DC probes. (**B**) Transmission through the devices labeled as neuron 1 to neuron 4, showing an insertion loss (IL) smaller than 10.8dB.

Figure 6A shows a schematic of one of the aforementioned plasmonic MZM-MZI pair. The unmodulated carrier enters the chip via a grating coupler and routed by a buried Si ridge waveguide (WG) to the plasmonic MZM. The light is first split into two arms by a 1 × 2 multimode interferometer (MMI). In each arm, the signal is coupled into a plasmonic slot WG by means of a photonic-plasmonic converter (*49*–*51*). After propagation through the plasmonic WG, the light is coupled back into a Si WG. The plasmonic slot was filled with an organic nonlinear material (NLM), a strong Pockels (*47*, *52*, *53*). Through the Pockels effect, the refractive index experienced by the optical field is changed by applying an RF voltage across the gold electrodes, resulting in a phase shift. In contrast to dielectric WGs, plasmonic WGs allow light to be confined into the subwavelength regime. This then results in a large RF field in the slot which overlaps well with the optical field. Consequently, those effects lead to a large phase change for a given voltage (*43*). This way, the slot length can be kept short. Concretely, the plasmonic 15-μm-long slot measures 105 nm in width. Not only allows this to fabricate compact device structures but also gives raise to the unprecedented bandwidth of up to 1 THz (*26*, *54*, *55*). Because of the short length, traveling-wave effects can be neglected, and the modulator can be modeled as a lumped capacitive load with a capacitance of a few fF. Consequently, the bandwidth and (device) energy consumption is very low (*28*, *29*, *55*, *56*). The MZM is operated in a ground-signal-ground configuration, where the RF field points in opposite directions in the two arms. To achieve intensity modulation through push-pull, the chromophores need to be aligned in the same direction in both slots. This alignment is set during a one-time poling process as described in (*47*). The MZM arms have an intentional length imbalance, introducing a static phase offset. This offset sets the operation point and can be adjusted by selecting a suitable wavelength. The phase-changed optical signals are then combined in a 2 × 1 MMI. The wavelength dependency follows the shape of the transmission spectrum in Fig. 6B. The wavelengths were selected at the 3-dB point, also known as the quadrature point, where one achieves best-performing intensity modulation.

After modulation, the signal is routed to a conventional T/O MZI that performs the weighting function. In contrast to the MZM, this MZI is balanced and thus shows negligible wavelength dependency. A heater is placed on top of the SiO_2_ cladding above one WG arm (see Fig. 6A, right inset). The heater, made mainly of gold, has a cross-sectional area of ~0.15 μm^2^ and requires about 23 mW to switch the power from full-on to full-off. The heater power was manually adjusted to match the pretrained ANN weights, thereby accounting for insertion-loss variations and any a priori uncertainties in the T/O MZI response. While in situ training was not used here, it could be a useful extension in future implementations to account for hardware drift or fabrication imperfections at a larger scale.

The device losses comprised the losses of the Si routings, T/O MZI, and plasmonic MZM, which are below 10.8 dB for each neuron. The transmission spectra of the asymmetric MZM are shown in Fig. 6B. The on-off voltages of the plasmonic MZMs were around 7.5 V_peak_. We estimate a bandwidth well above 100 GHz. We justify this estimation by pointing toward previous bandwidth measurements of the PO platform in various electrode and modulator designs (*26*, *27*, *54*, *55*, *57*, *58*). Those designs include compact and large electrodes as well as MZM, ring, and racetrack configurations. Following (*28*), the energy consumption per bit was estimated to be 15.3 fJ/bit. Thereby, we assumed a uniformly distributed two-level signal and a capacity of 3.4 fF. The estimation of the capacity value is in line with the bandwidth estimation and is therefore typical for a PO modulator; also as reported in the previous works (*28*, *29*, *55*). The active section of the plasmonic phase shifter is only 7.5 μm^2^. A 170 μm–by–192 μm rectangle can enclose the Si routings for the MZM. Note that on-off voltages and device losses are typically much lower in devices fabricated using Polariton’s current PDK, compared to (*57*, *59*, *60*). Such device parameters would, for example, allow for a reduction in the driving voltage by a factor of 3 down to 1 V_peak-to-peak_. This would lead to a power consumption of only 0.85 aJ/bit. Also note that plasmonic modulators can also be as compact as π (1)^2^ μm^2^ (*27*) and can feature insertion loss below 2 dB (*48*, *58*).

**Fig. 7.**
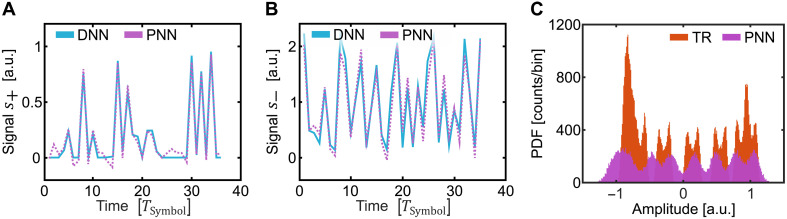
Trace details and symbol histogram. (**A** and **B**) Signal traces for $s_{+}$ and $s_{-}$, respectively, with the expected DNN trace in blue and the recorded PNN trace in pink. (**C**) Histogram of the recoded TR and PNN signal. The histogram represents a probability density function (PDF) estimated in counts per bin.

The PNN performance was then tested. Toward this end, we processed the 48 Gbit/s 8PAM signal of the first and second neural network layers offline. The third layer was then executed on the plasmonic-enhanced optical accelerator. The traces in Fig. 7 (A and B) show measured values of a sequence as calculated by the PNN (violet). To verify the operation, we compared traces of the sampled data at the output of the third layer with the respective sequence of an equivalent DNN implementation (solid blue lines). One can see that the measured and expected values match reasonably well. This indicates that the weight setting was sufficiently accurate. The histogram in Fig. 7C showcases the effect of the PNN. The red and violet bins represent the signal distribution of the 8PAM signal after TR and after the PNN, respectively. One can see that the TR signal has five of eight signal levels distinctly separated, while the remaining levels appear compressed. The PNN is able to undo this nonlinear distortion more effectively.

Equalizing around 10^5^ symbols with the PNN translates to an SNR performance of 16.1 dB as shown Fig. 3A. Compared to the DNN, the analog execution results in lower SNR. However, the PNN outperforms the digital FFE by 1.6 dB in SNR. In terms of bit error ratio (BER), the usage of the PNN decreased the BER to a value of 3.8 × 10^−2^. The PNN thus pushed the BER below the soft-decision forward-error-correction threshold. The FEE only decreased the BER to a value 8.0 × 10^−2^. It should be stressed that this first implementation of the photonic accelerator was performed with a device that leaves room for improvement. When simulating the PNN with hypothetical devices that feature typical on-off voltages and typical insertion losses, one could markedly increase the performance of the PNN. Concretely, assuming an on-off of 3.1 V_peak_ and edge coupling losses of 1.0 dB, one can show that with PNN one should be able to reach 23.1 dB in SNR, see Fig. 3B (pink empty circle).

The number of multiplications for the PNN has been given with around 30 in the plot of Fig. 3B. These multiplications are needed to execute the first and second layers in the digital domain. This is by all means a low number of operations. It can therefore be envisioned that the whole DNN can be fully realized within an all PNN. Simulating the achievable SNR as executed by such a PNN (relying on realistic MZM and MZIs) would lead to an SNR of 21.2 dB without a need for a single digital operation, see Fig. 3B (full pink circle). This finding indicates that the PNN has the potential to surpass even advanced digital nonlinear equalizers, such as the VLT series, while basically requiring no digital multiplications. Such a PNN then would only require the dc power to tune the weights, to operate the detectors, and to operate the MZMs. The weights could alternatively be tuned by piezoelectric, electro-optical, or efficient T/O phase shifters, e.g., (*61*–*63*), respectively. The photodetectors are reverse-biased PDs with a low power consumption, and the plasmonic MZMs can be operated with very low voltages, which makes a plasmonic realization most attractive.

### Performance comparison with the state of the art

**Fig. 8.**
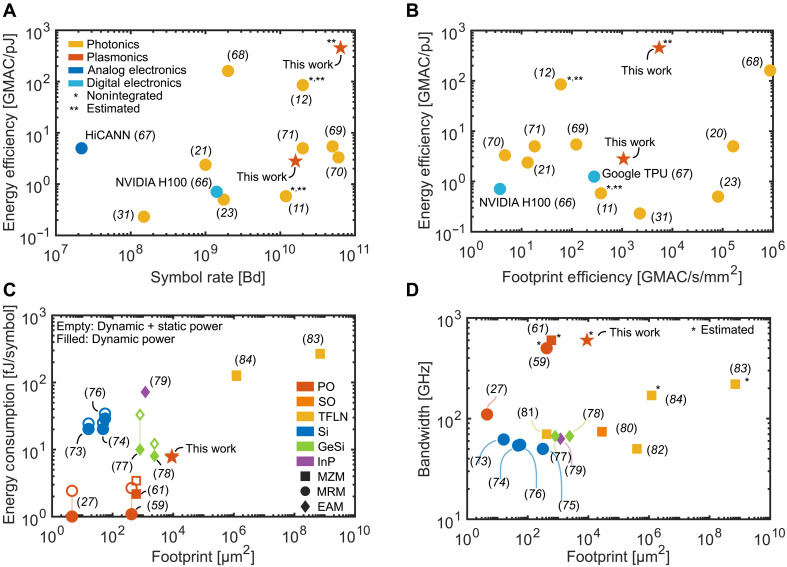
Comparison of plasmonic technology versus state of the art. (**A**) Energy efficiency (GMAC per petajoule) as a function of symbol rate (Baud); (**B**) energy efficiency versus footprint efficiency (GMAC per second per square millimeter); In (A) and (B), the data include our work (red star), photonic neural network (yellow), analog electronic (dark blue), and digital electronics (light blue). The estimated (**) performance indicates the potential of our work. We also included recent nonintegrated, i.e., not on-chip, approaches (*) and estimated (**) their performance when it would be integrated on a photonic platform. (**C**) Modulators’ energy consumption per symbol (femtojoules per symbol) versus device footprint (μm^2^). The energy consumption of the modulator was estimated by taking only dynamic power consumption (filled marker) or the additional static power (empty marker) including electronic and optical contribution; (**D**) analog bandwidth (gigahertz) versus footprint (square micrometers). (*) denotes that the bandwidth was either estimated or extrapolated. In [(C) and (D)], the data include our work (red star), recent PO modulators (red), photonic-oganic (SO) modulator (orange), silicon photonic modulators (blue), thin-film lithium niobite (TFLN) modulators (yellow), indium-phosphate (InP) platform (violet), and germanium-silicone (GeSi) platform (green). The box, circle, and diamond represents MZM, microring modulators (MRMs) and electro-absorption modulators (EAMs), respectively.

In this section, we benchmark our PNN and the underlying PO modulators against the state of the art in photonic neural network accelerators and optical modulator technologies. For neuromorphic systems, we evaluate faithfully performance in terms of energy efficiency (GMAC per petajoule), footprint efficiency (GMAC per second per square millimeter), and symbol rate (Baud). In Fig. 8 (A and B), we show the energy efficiency against the footprint efficiency and symbol rates at which recent system operates, respectively. Analog (HiCANN) and digital (NVIDIA H100, Google TPU) electronic systems are shown as an additional reference point (*64*–*66*). While many recent systems achieve impressive total throughput, often exceeding 10^9^ operations per second (1 TOPS), often exceeding 1 TOPS) and high levels of integration with electronics, their operating speeds typically remain below 2 GHz (*21*, *23*, *31*, *67*). For the online processing of signals at symbol rates exceeding 100 GBd, a slow operational speed necessitates the parallelization of the workload, resulting in an increase in latency. A small latency is crucial in inter- and intradatacenter, as well as intrarack, links. Our current implementation operates at 16 GBd. However, given the bandwidth of the modulators and the analog photonic nature of our architecture, we project that operation at ≥64 GBd is feasible. On the basis of this projection and assuming full optical implementation of all three layers, we estimate an energy efficiency of 450 GMAC/pJ and a footprint efficiency of 5500 GMAC/s per mm^2^ (red star with **). For reference, the current prototype achieves ~30 GMAC/pJ and ~900 GMAC/s per mm^2^. The latency of our approach will be dominated by the time the signal required to propagate through the delay lines. Yet, this introduced delay cannot be avoided. Recent integrated approaches have already demonstrated higher operational speeds exceeding 10 GHz while achieving throughputs below 1 TOPS (*68*–*71*). Demonstrations of nonintegrated approaches (marked with *) with operation speeds above 10 GHz and above 1 TOPS have already shown the potential (*11*, *12*). Integrated approaches at speeds above 10 GHz have so far relied on Si modulators, GeSi electro-absorption modulators (EAMs) and thin-film lithium niobate (TFLN) MZM. To our knowledge, we demonstrate the first neural network architecture employing plasmonic modulators. The potential of the plasmonic approach is ideal as it allows efficient and fast modulation at a small footprint. To illustrate this, we compare the footprint, bandwidth, and energy consumption of TFLN, Si, GeSi, silicon-organic (SO), and indium-phosphate (InP) against the PO platform.

Figure 8C shows the energy consumption of the modulators against the modulator footprint. Si microring modulators (MRMs), shown in blue, consume little power around 20 fJ per symbol at a very small footprint of around 25 μm^2^, making them ideal for tight packing (*72*–*75*). GeSi EAMs (green) require slightly more space while having a smaller dynamic power consumption associated with the modulation (*76*, *77*). InP EAMs have a similar footprint to their GeSi counterparts but have a larger energy consumption (*78*). Taking the static power consumption into account, EAMs need energy in the same order of magnitude than Si MRMs. TFLN MZMs require a large footprint as they typically have a length of >1 mm. Because of their length, the power consumption is also substantially increased compared to the other approaches. PO MZMs have a similar footprint to EAMs but require around 10 times less power (*57*). The footprint and energy consumption (due to halving the capacity) can be further reduced by using MRM structures (*27*, *58*), with record-low footprint of 2π (1 μm)^2^ and low energy consumption of <10 fJ per symbol. We conclude that only PO modulator techniques have the potential for small energy consumption at the lowest footprint.

Figure 8D shows the bandwidth of the modulator technologies. One can see that Si MRM and EAMs and SO MZMs have a bandwidths below <70 GHz (*79*). Only PO and TFLN show bandwidths above 100 GHz; however, TFLN needs to trade in footprint for higher speeds (*80*–*83*). On the other hand, PO modulators have shown bandwidth up to 1 THz, yet, here, we assume slightly reduced bandwidth (600 GHz), as the pads are slightly larger than in (*55*). We conclude that plasmonic modulators uniquely combine a small footprint and high bandwidth. Plasmonic modulators are so far the only type capable of transmitting 160 GBd at 8PAM signaling over simple IM/DD, requiring linear DSP methods, which indicates good signal quality at high resolution and speed.

Device-level characteristics are shown in Fig. 8 (C and D). Figure 8C displays energy consumption per symbol versus device footprint, distinguishing between dynamic-only (solid markers) and total power including static contributions (open markers). Our modulator occupies a favorable position, combining subfemtojoule energy per symbol with minimal footprint. Figure 8D shows analog bandwidth versus footprint, where our design is estimated to achieve more than 100-GHz bandwidth within a sub-100-μm^2^ area. These results already point toward the potential of using PO modulators for scalable, low-latency, and high-throughput photonic neural processing.

### Performance in a fiber transmission

**Fig. 9.**
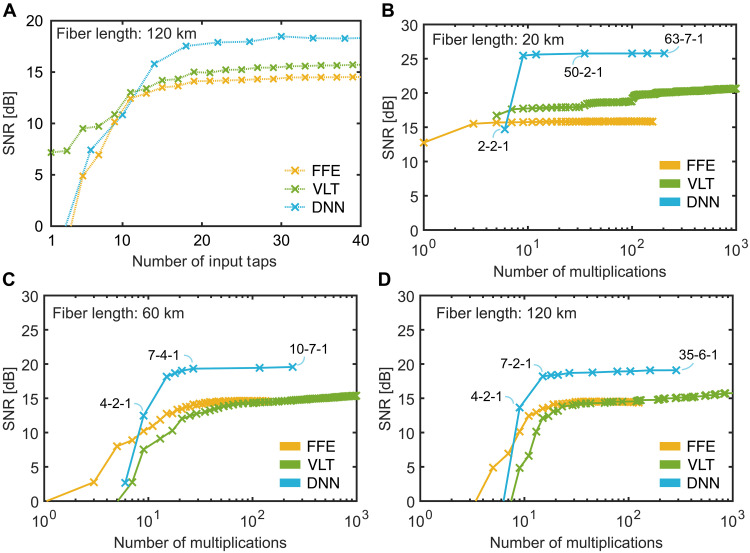
Fiber transmission. (**A**) SNR after DSP with an FFE (yellow), VLT (green), and the DNN (blue) for swept numbers of input taps. The signal propagated in simulation through a 120-km-long standard single mode fiber. (**B**) SNR after DSP with an FFE, VLT, and DNN reported for the required number of multiplications. In contrast to (A), in (B) to (D), all the higher-order taps of the VLT and the hidden and output layer size are swept on top of different fiber lengths: 20 km (B), 60 km (**C**), and 120 km (**D**). The annotations describe the network architecture.

In the last step, we explore the generalization potential of the DNN concepts beyond the initial channel conditions. Specifically, we test the performance when transmitter and receiver are separated by a long fiber link. In this case, the equalizer must not only handle nonlinear distortions of the amplifier but also compensate for signal degradation originating from power fading due to chromatic dispersion (*33*, *84*). This scenario demonstrates that the neural network can be adapted to various channel worsenings and still be more effective and efficient than traditional FFE and VLT schemes. Toward this end, we picked a realistic scenario of a 120-km fiber link operating at 48 Gbit/s with PAM8. To compensate for the dispersion, we increased the number of taps. Intuitively, one would expect that dispersion compensation requires a higher number of tap delays. In practice, we increased the number of the first-order taps in the FFE and VLT scheme. The number of taps in the VLT’s second and third orders was fixed at 21 and 9 taps, respectively. For the DNN, we increased the number of neurons in the input layer. The number of neurons in the ANN’s hidden layer was kept at four. The required number of input taps in an FFE or VLT, respectively, and the numbers of nodes in the input layer of the DNN for a given SNR are shown in Fig. 8A. It can be seen that the FFE, VLT, and DNN can cope with a 120-km-long standard single mode fiber that—on top of the electrical amplifier—distorts the signal. From the plots in Fig. 9A, one can see the following: First, as expected, all three methods require a higher number of taps to converge to plateaued SNR values when comparing the results with those in Fig. 3. Second, although the overall performance is decreased, the DNN outperforms both the FFE and VLT DSP. It plateaus at a higher SNR for a relatively low number of input taps.

Figure 9 (B to D) shows the SNR with respect to the number of multiplications. For those plots, we swept not only the number of input taps but also the taps of the second and third VLT orders as well as the number of nodes in the hidden layer. We then confined ourselves to plotting the Pareto front, i.e., the best SNR values achieved, with the respective multiplications. We investigated the performance for fiber lengths of 20 km (Fig. 9B), 60 km (Fig. 9C), and 120 km (Fig. 9D). The annotations describe the network architecture of the form of P-H-1, where P is the number of input taps and H the number of hidden neurons. One can see that increasing the fiber length results in a worsening of the SNR. The distortion cannot be fully recovered by the algorithms. Yet, the DNN achieves a larger SNR compared to the FFE and VLT throughout all investigated lengths. This is obtained by a similar number of multiplications. Already with as little as 30 multiplications, one can outperform the operation of a traditional DSP. It is worth to highlight that the simulations give an idea about the flexibility of the DNN architecture to be able to adapt itself to different channels. This flexibility comes in handy when on-the-fly adjustment to its weights via a common offline feedback. As the DNN has only a small weight count, those adjustments can be achieved efficiently.

It can be concluded that a DNN solution already now can outperform traditional DSP processing in reasonably medium- and short-haul transmission links. It can be seen that an optical on-chip PNN approach where calculations are performed in the analog domain becomes within reach. The low number of multiplications in the order of 30 shows that PNN may soon become practical.

## DISCUSSION

The comparative analysis of digital and photonic signal processing techniques for mitigating nonlinear distortions in optical fiber communication systems has demonstrated clear advantages of ANNs over traditional DSP methods such as FFE and VLT series. The DNN solution showcased superior performance, achieving higher SNR at the price of lower computational complexity. It has been shown that DNN solutions with as little as 30 MACS are sufficient to handle nonlinear distortions and dispersion for distances of up to 120 km. This work confirms that DNNs outperform traditional equalizers and moves beyond prior research by demonstrating a PNN implementation of the DNN featuring plasmonic modulators capable of nonlinear channel equalization with very few nodes. PNNs present a compelling alternative, leveraging the inherent properties of light to process information more efficiently. The study demonstrated that PNNs, particularly when using PO-hybrid MZMs, can substantially enhance computational speed, reduce power consumption, and reduce footprint. Although the SNR performance of PNNs was lower than that of DNNs, they still outperformed the traditional methods TR and FFE and offered substantial complexity reductions. The experimental results confirmed the potential of PNNs in processing high-speed optical signals, achieving an SNR improvement over conventional linear digital filters. In light of the low number of MACs required for signal processing in ANNs, the integration of photonic elements within ANN architectures indicates a promising future for optical signal processing, characterized by reduced power consumption, increased processing speeds, and enhanced SNR.

In conclusion, this study highlights the transformative potential of photonic-plasmonic computing in optical communications and demonstrates how the advantages of DNN can be translated into PNNs, which pave the way for their adoption in next-generation communication systems.

## MATERIALS AND METHODS

### Training and test data acquisition

Data for training and testing the ANN were acquired using a 100-GSa/s AWG that generated symbols in an 8PAM format at a symbol rate of 16 GBd. An electrical amplifier introduces nonlinear distortions and drives a 30-GHz photonic modulator with an on-off voltage of 3 V_pp_. After an EDFA, the optical signal was filtered and converted in a 70-GHz PD and sampled with a 160-GSa/s real-time oscilloscope. The data after the TR were resampled to two samples per symbol and used for training. Thereby, the time between samples corresponds to the time delay from the envisioned delay line.

The ANN was trained using the back-propagation algorithm with custom code written for MATLAB. The network consisted of seven input nodes, four hidden neurons with sigmoid activation, and one linear output neuron. The choice of the typically used sigmoid activation is not essential, as the network performs similarly with other activation functions, such as the rectified linear unit (ReLu). During training, an ideal sigmoid function was used. No experimental PD characteristics or other nonidealities were taken into account. For training, around 100 epochs were used at a learning rate of 0.05. The train-test split was 10:90. The number of epochs and the learning rate were chosen empirically to ensure stable and fast convergence. The 10% training share corresponds to approximately 10^4^ symbols, which helps avoid overfitting and aligns with the training ratio used for the other equalizers. In Table 1, we summarize the above training setup and main parameters used for the ANN.

**Table 1.** Details on DNN architecture and its training. First column presents the parameter whose value is given in the second column. In the third column comments, explanations or rational is presented.

Parameter	Value	Notes
Network architecture	7-4-1	Seven inputs, four hidden, one output neuron
Activation function	Sigmoid/linear	At hidden/output layer
Training algorithm	Backpropagation	Custom implementation in MATLAB
Learning rate	0.05	Chosen empirically for stable and fast convergence
Training epochs	~100	Sufficient for convergence
Train/test split	10%/90%	Same split used for other equalizers
Training data size	~10^4^	Avoids overfitting

### Experimental setup of the PNN

In the experiment, the first and second layers of the neural network were processed offline, while the third layer was realized on-chip. For the experiment, fabrication penalties, such as insertion loss and delay line delay, were not considered in the offline processing. The optical carriers were generated using four tunable laser sources. The wavelengths were selected such that each adjacent frequency difference is much larger than 100 GHz and such that the quadrature point of the MZMs does not need to be reached with active biasing. Setting the operation point by wavelength is possible since the arm length of the MZM was designed to be different, resulting in an MZM spectrum as shown in Fig. 6. Concretely, we selected the wavelengths 1551.54, 1550.17, 1547.3, and 1548.94 nm for neurons 1 to 4, respectively. The in and out coupling was performed using a nonpolarization-maintaining multicore fiber and grating couplers. The T/O heaters were contacted via a GSGSGSGSG dc probe wedge and used to set the optical power to match the pretrained weights by sequentially measuring the optical power at each output. In this way, also imperfection such as variations in insertion losses were compensated, and a priori uncertainties in the set weight values were accounted for. The four plasmonic MZMs were connected to a 100-GSa/s AWG with four outputs via a GSGSGSGSG RF probe wedge probe (67 GHz). No packaging was implemented. The optical signals were then combined and amplified as outlined here in the main text and detected by two PDs and sampled with a 160-GSa/s real-time oscilloscope (RTO).

### Assessments of processing demands

The number of multiplications shown in Fig. 3B was used as a simple metric to estimate the computational processing demand of each method. For the DNN, FFE, and VLT schemes, this corresponds to the actual number of digital multiply operations per symbol as detailed in the “Feed-forward equalizer” section. The reduction in processing demand when switching from digital methods to the PNN is represented by the number of multiplications that were shifted to the optical domain and are no longer required digitally. The lower multiplication count for the PNN does not imply a full system-level energy comparison but rather illustrates the reduced reliance on electronic digital processing demands in layers executed optically.

To generate the full pink circle in Fig. 3B, we performed a simulation of an entirely optical implementation, assuming that all layers were realized on a photonic or plasmonic platform. No digital multiplications were used in this case. Imperfections were introduced by accounting for an electrical amplifier and mixing effects in the photodetector. The electrical amplifiers are assumed to have a noise figure of 5.5 dB, representing the dominant electronic noise contribution. The photodetection was modeled as square-law detection with a responsivity of 0.6 A/W, which accounts for both signal-noise and noise-noise mixing effects. The optical input power to each of the photodetector is assumed to be −3 dBm.

### Energy consumption of modulators

To calculate the energy consumption of the modulators, we calibrated the reported values to a two-level driving signal. For the static energy consumption, we considered the electronic contributions of 1 mW for EAMs, 100 μm for Si MRMs, and 0 W for TFLN and PO MZMs, as well as the optical contributions, which included the device’s insertion loss and modulation losses. The modulation losses were 2 dB for EAMs, 6 dB for MRMs, and 3 dB for MZMs. Then, the energy was calculated by assuming 10 mW of optical power for the duration of a symbol at the bandwidth limit. We thereby clipped above 100 GHz.
