# Supplementary material for: Digital and plasmonic artificial neural networks—Improved nonlinear signal processing at high speed and low complexity
Source: Sci Adv. 2025 Nov 14;11(46):eadx1657. doi: 10.1126/sciadv.adx1657 (PMC12617482; doi:10.1126/sciadv.adx1657)
Supplement: Supplementary file 1 — Supplementary Text Tables S1 and S2 [file sciadv.adx1657_sm.pdf]

Science Advances
AAAS

Supplementary Materials for

Digital and plasmonic artificial neural networks—Improved nonlinear signal processing at high speed and low complexity

Tobias Blatter *et al.*

Corresponding author: Tobias Blatter, tobias.blatter@ief.ee.ethz.ch; Juerg Leuthold, juerg.leuthold@ief.ee.ethz.ch

Sci. Adv. **11**, eadx1657 (2025)
DOI: 10.1126/sciadv.adx1657

This PDF file includes:

Supplementary Text
Tables S1 and S2

Supplementary Text

Details on power consumption calculations

To calculate the power consumption we followed Reference (*30*).

$$P_{\text{consumed}} = N_i \frac{P_{\text{R}}}{\eta_{\text{wp}}} 10^{-\alpha[\text{dB}]/10} + RN_i \cdot E_{\text{mod}} + N_i N_{i+1} P_{\text{weight}}$$

where P_{consumed} is the power consumed of the ith layer. The first term is the total wall-plug laser power required to meet the sensitivity of the PD P_{R}, and depends on the photonic and plasmonic losses α and wall-plug efficiency η_{wp}. Note that for the first layers, we assume that there is no additional laser power required. The second term is the power consumed by the neurons. Here, R and N_i is the symbol rate and the number of neurons in layer i. The last term represents the power consumed by the weight. Here, P_{weight} is the average power consumed by the weighting element.

The Table S1 summarizes the variables assumed to calculate the power consumption of the plasmonic ANN.

The Table S2 summarizes the variables assumed to calculate the power consumption of the plasmonic ANN chip in the projected case in which all layers including the delay section are implemented on-chip.

Table S1.

Parameters to calculate the power consumption of the chip as in experiment.

η_{wp}	10%	
R	16 GHz	Experimental condition
N_2	4	Hidden Layer
N_3	1	Output Layer
E_{mod}	15.3 fJ	$E_{\text{mod}} = \frac{1}{2} V_{\text{pp}}^2 C_{\text{MZM}}$
V_{pp}	3 V	Driving Voltage
C_{MZM}	3.4 fF	Estimate from (*55*)
$V_{\text{PD,bias}}$	2 V	Experimental condition
P_R	0.04 mW	Experimental condition
α	11 dB	Measured average power on-chip loss
P_{weight}	$\frac{1}{4}$23 mW	Measured P_π = 23 mW

Table S2.

Changed parameters to calculate the power consumption of the chip for extrapolation.

R	64 GHz	Experimental condition
N_1	7	Input Layer

E_{mod}	2.2 fJ	$E_{\text{mod}} = \frac{1}{2} V_{\text{pp}}^2 C_{\text{MZM}}$
V_{pp}	1.6 V	Typical driving voltage
C_{MZM}	$\frac{1}{2}$3.4 fF	Ring requires only one phase shifter
P_{weight}	$\frac{1}{4}$100 nW	BTO Phase shifter

Comparison between VLT and ANN

Here, we explore the relationship between VLT and ANN. We compare the VLT and ANN by sketching out that an ANN can be represented by an infinite VLT series. Since an infinite VLT series is arguably hard to calculate, this shall provide an example why ANN can be computationally cheaper. We thereby follow Reference (*39*).

Let's say that $\{z(t), z(t-1), \dots, z(t-n)\}$, represents N tapped-delayed inputs to the NN, v_{ji} the weights from the input delay j in the hidden neuron i, b_i the bias of the neuron i in the hidden layer, σ an non-polynomial, analytic activation function and w_i the weights from the hidden neuron i to the linear output. The output $y_{NN}(t)$ of a NN with one hidden layer with M neurons and a linear output layer is given by

$$y_{NN}(t) = \sum_{i=1}^{M} w_i \sigma\left(\sum_{j=0}^{N} v_{ji}\, z(t-j) + b_i\right).$$

Through the Universal Approximation Theorem, such a network can approximate any function. Here, the function is the inverse of the channel with a memory smaller than N. By definition of σ, we can write it as an infinite Taylor series, i.e.

$$\sigma_i(x) = \sigma(x + b_i) = \sum_{n=0}^{\infty} \alpha_n^{(i)} x^n.$$

We plug in the term $x = \sum_{j=0}^{N} v_{ji}\, z(t-j) + b_i$ and expand the n-th power term, i.e.

$$\left(\sum_{j=0}^{N} v_{ji}\, z(t-j)\right)^n = \sum_{m_1=0}^{N} \dots \sum_{m_n=0}^{N} v_{m_1,i} \dots v_{m_n,i} \cdot z(t-m_1) \dots z(t-m_n).$$

To convince oneself one can proof the expansion through induction over n. With the above equation, we can rewrite the NN output as

$$y_{NN}(t) = \sum_{i=1}^{M} w_i \sum_{n=0}^{\infty} \alpha_n^{(i)} x^n$$

$$= \sum_{i=1}^{M} w_i \sum_{n=0}^{\infty} \alpha_n^{(i)} \left[\sum_{m_1=0}^{N} \dots \sum_{m_n=0}^{N} v_{m_1,i} \dots v_{m_n,i} \cdot z(t-m_1) \dots z(t-m_n)\right]$$

Without proof, we swap the order of the summation terms and arrive at

$$y_{NN}(t) = \sum_{n=0}^{\infty} \sum_{m_1=0}^{N} \dots \sum_{m_n=0}^{N} \left[\sum_{i=1}^{M} w_i \alpha_n^{(i)} v_{m_1,i} \dots v_{m_n,i}\right] \cdot z(t-m_1) \dots z(t-m_n).$$

This takes now the form of an infinite VLT series. Their kernels are given by the term in the square bracket. We now can argue that this is hard to calculate as it involves infinite many discrete calculations. On the other hand, an ANN is still relatively cheap to calculate. However, it is important to note that this is not a general proof that ANN is always easier to calculate. In fact, one can reverse the argument: For a finite VLT series, up to order P and kernels h, i.e.

$$y_{VLT}(t) = \sum_{p=0}^{P} \sum_{m_1=0}^{N} \dots \sum_{m_p=0}^{N} h_p(m_1, \dots, m_p) \cdot z(t - m_1) \dots z(t - m_p),$$

one would run into the problem that one would need to set $\alpha_n^{(i)} = 0$ for $n > P$. This contradicts the assumption that the ANN is non-polynomial. However, one can set the activation function as required. Without proof, we assume that it is always possible to find the $\alpha_n^{(i)}$ (they still differ through the bias) such that the required Taylor series can be constructed. This then would lead to the same performance yet may increase computational effort by requiring explicit, coefficient-wise evaluation of unoptimized Taylor coefficients. To investigate the latter, we assume that $P < \infty$ and all kernels are neither 0 nor 1 and the VLT perfectly represents a channel. The question then is how complex would the network be in order to generate the same output as the VLT. Note that a perfect VLT representation is a strong assumption since it is very unlikely that a real-life channel can be perfectly represented with a finite polynom. Then, the number of multiplications for the VLT is

$$\sum_{p=0}^{P} p \binom{p+N}{p}.$$

Thereby, we ignored symmetries that would help reducing the number of multiplications. On the other hand, the number of multiplications of the ANN (including the activation function given through the Taylor series calculated using Horner's method) is

$$M(N + P + 1).$$

The question remains how big M, i.e. how many neurons one would need.
